# Velocimetry of superconducting vortices based on stroboscopic resonances

**DOI:** 10.1038/srep35687

**Published:** 2016-10-24

**Authors:** Ž. L. Jelić, M. V. Milošević, A. V. Silhanek

**Affiliations:** 1 Département de Physique, Université de Liège, Aleé du 6-Août 19, B-4000 Liège, Belgium; 2 Departement Fysica, Universiteit Antwerpen, Groenenborgerlaan 171, B-2020 Antwerpen, Belgium

## Abstract

An experimental determination of the mean vortex velocity in superconductors mostly relies on the measurement of flux-flow resistance with magnetic field, temperature, or driving current. In the present work we introduce a method combining conventional transport measurements and a frequency-tuned flashing pinning potential to obtain reliable estimates of the vortex velocity. The proposed device is characterized using the time-dependent Ginzburg-Landau formalism, where the velocimetry method exploits the resonances in mean vortex dissipation when temporal commensuration occurs between the vortex crossings and the flashing potential. We discuss the sensitivity of the proposed technique on applied current, temperature and heat diffusion, as well as the vortex core deformations during fast motion.

The resistive state of superconducting thin films in an external magnetic field and applied transport current is a consequence of the dissipative motion of vortices^1^,^2^. While the slowly moving Abrikosov vortices retain their cylindrical core, it has been theoretically shown that vortices traveling at high velocities exhibit core deformation and tailgating of quasiparticles (for recent studies of the behavior of vortex core quasiparticles under applied current see Ref. 3,4), due to which moving vortices tend to align and connect to the wake of the preceding vortex^5^. Such elongated vortices are often referred to as Abrikosov-Josephson, resembling the ones found at step-edges and grain boundaries^6^,^7^,^8^,^9^,^10^. Their further acceleration in increasing current leads to the formation of a phase-slip^11^, a line of suppressed superconductivity across which the phase of the superconducting order parameter changes by 2*π*, and along which phase singularities move with high velocity. Those coreless yet moving singularities are often referred to as Josephson vortices (in analogy to those in S-N-S junctions^12^,^13^,^14^,^15^).

Real-time observations of the vortex motion are notoriously difficult to achieve, because vortex velocity in the condensate largely exceeds the sweeping rate in most of the scanning probe techniques and the time between two consecutive frames in snapshot techniques^16^,^17^. Estimates of the average vortex velocity reported in literature have been mainly obtained from transport measurements^18^,^19^,^20^. Relative vortex velocity, and vortex-vortex interaction within the dynamic regime have also been studied in the past, using the Corbino setup^21^. Even the dispersion of the vortex velocities has been measured^22^. All of the obtained results concurred that the speed of the Abrikosov vortices can be as high as several km/s, while the velocity of Josephson vortices can rise to 100 km/s^23^.

The investigation of magnetic flux dynamics in superconductors by means of (passive) stroboscopic effects has been of experimental interest since the late 1970’s^24^. More recently, theoretical investigations of motion of the flux patterns under periodic light illumination predicted (active) stroboscopic resonances in resistance and voltage when the excitation frequency matches the frequency of the characteristic vortex dynamics^25^. Following the same prescription, in this paper we devise a concept for vortex velocity measurement stemming from the stroboscopic states in a superconductor with a dynamic pinning landscape. We show that this approach, in which we take into account spatial and temporal heat dissipation, is able to precisely capture vortex velocities, even very high ones, and thereby discern different vortex phases at the crossover from Abrikosov to Josephson ones, each of which are tunable either by external modulation, or by temperature, current, or magnetic field.

## Results

**Figure 1:**
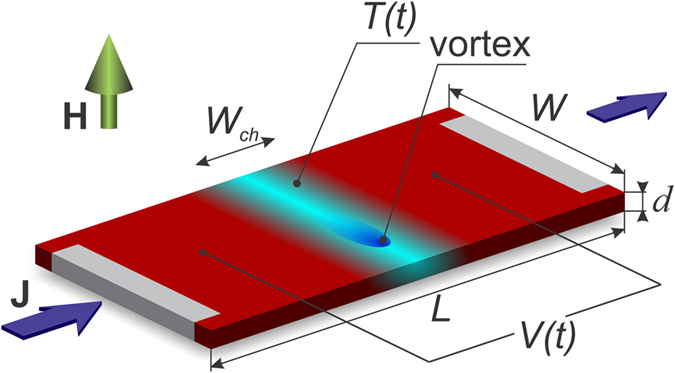
The oblique view of the investigated system. Superconducting bridge (of size *L* × *W*) is exposed to a perpendicular magnetic field **H** and longitudinal transport current density **J**. In the center of the bridge a channel of depleted superconductivity (of width *W*_*ch*_) is created by local heating, and varies over time. Time-dependent voltage *V*(*t*) is measured across the channel.

We consider a thin superconducting film (of size *L* × *W*, with thickness *d* ≪ *λ*, *ξ*, where *λ* and *ξ* are the field penetration depth and superconducting coherence length, respectively), exposed to an external magnetic field *H*, and *dc* transport current density *J*, which exhibits recurring stroboscopic resonances when exposed to external potential that periodically suppresses and recovers superconductivity in the channel (of width *W*_*ch*_) across the specimen, as depicted in Fig. 1. Such time-periodic potential can be achieved in several ways. For example, mode-locked solid-state lasers can emit pulse repetition rates between 50 MHz and a few gigahertz^26^ (in extreme cases above 100 GHz), and can modulate the superconducting condensate by localized heating. Note that in order to confine light to few tens of nm (corresponding to the size of the channel in our work), one can envisage to use nanoscale metallic waveguides which transform the long wavelength incoming light into surface plasmon polaritons able to be focused down to the required scales^27^. Recently demonstrated nano-heating source^28^, using a single Ag nanowire as resistive nano-heater and where possible bandwidth can be tuned by the pulsed current, could possibly be used to locally deplete the superconducting condensate periodically. Alternatively, one can envisage the use of low-temperature scanning electron microscopy^29^, operating down to 4 K, where electron beam sizes of few tens of nm are equipped with fast electrostatic beam blankers with rising time of sub-ns and repetition rate of 300 MHz.

As a representative example we use parameters for NbN thin films^30^ [*ξ*(0) = 4.2 nm being the coherence length at 0 K, the critical temperature *T*_*c*_ = 12.7 K, and the normal state conductivity *σ*_*n*_ = 4241 S/cm] and the sample size of *L* × *W* × *d* = 400 × 100 × 6 nm^3^. Bath temperature is set to *T*_0_ = 0.9*T*_*c*_, to respect the validity domain of the employed time-dependent Ginzburg-Landau (TDGL) theory^2^, and applied field is *H* = 90 mT. Using the TDGL simulations^2^,^31^ (see Methods) we reveal the dynamic response of the sample to the applied current in the presence of an external magnetic field and a periodic confining potential across the sample, by calculating the temporal evolution of the spatial profile of the superconducting order parameter Ψ(**r**, *t*), the Cooper-pair density |Ψ(**r**, *t*)|^2^ and the electrostatic potential *φ*. For the pulsating potential we use the temporal oscillation of local temperature, hence we also take into account the diffusion of heat in the system. For the latter, the equation of thermal balance^32^ is employed to monitor the spatial and temporal evolution of the temperature *T*(**r**, *t*):


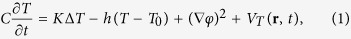


where parameters *C*, *K*, and *h* are the dimensionless heat capacity, heat conductivity and heat-transfer coefficients, respectively (see Methods for details). (∇*φ*)^2^ represents the Joule losses generated by the normal component of the current density, and *V*_*T*_ is the excess heat generated by the external source, modelled by 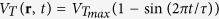 inside the channel of width *W*_*ch*_ = 5*ξ*(0), and 0 outside (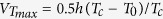 is the amplitude of the potential, and *τ* is the period of the oscillations).

**Figure 2:**
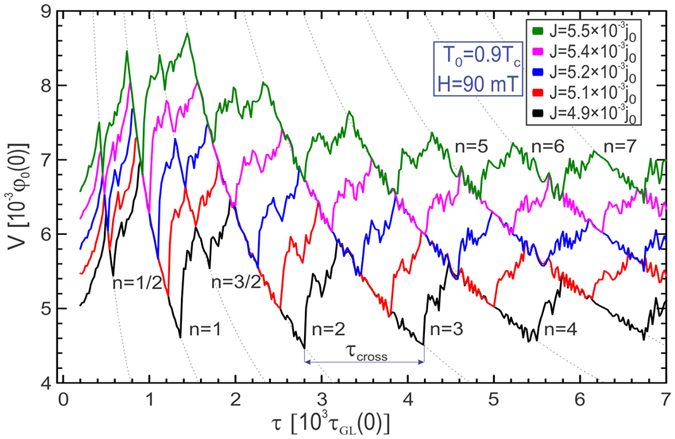
Stroboscopic voltage resonances. Quasi-periodic voltage plotted versus period *τ* of the oscillating thermal channel shown in Fig. 1, for given magnetic field and temperature, and for five different values of the applied current. The inter-resonance period is denoted as *τ*_*cross*_. Dotted lines indicate the 1/*τ* profile of the voltage during the resonances. Numbers *n* = 1−7 indicate the order of the resonance. Fractional resonances are also visible, and denoted by fractional *n* numbers. In the Supplementary Materials we present animations of the condensate behavior corresponding to the first four resonances during one period *τ* of the characteristic dynamics of the condensate (entitled Supplementary-Animation1.gif).

The calculated time-averaged voltage as a function of the channel flashing period [*V*(*τ*)] is shown in Fig. 2 for five values of applied current density *J*. As the channel transits between hot (ON) and cold (OFF), and vice versa, the superconducting order parameter in the channel is being suppressed and recovered, respectively, creating a favorable path for vortices to travel across the sample. The order parameter Ψ(**r**, *t*) relaxes over a finite time, of the order of the Ginzburg-Landau time *τ*_*GL*_(0)/(1 − *T*/*T*_*c*_) (see Methods), and is also influenced by the pinning potential period, *τ*, which in our simulations is at least one order of magnitude larger than *τ*_*GL*_(0). Because of these competing time-scales, it is obvious that thermal variations will not cause instantaneous changes in Ψ(**r**, *t*). In fact, the response of the condensate in the channel depends on the thermal diffusion of the heat supplied by the time-dependent pinning potential. The time over which the thermal diffusion becomes relevant is proportional to the ratio *C*/*K* in Eq. (1). In principle, the parameters used to calculate heat diffusion (thermal capacity, conductivity and heat transfer coefficient) are temperature dependent^33^ and also depend on the substrate properties (e.g. thickness of the substrate *d*_*s*_, heat capacity *c*_*s*_, and heat conductivity *k*_*s*_ of the substrate). For example, in the refs 32,34 effective heat capacity *c_f_  + d_s_ c_s_/d* and effective heat conductivity *k_f_  + d_s_ k_s_/d* are used (_c_f__ and *k_f_* are the real-unit heat capacity and heat conductivity of the film, respectively), with heat transfer coefficient *k_s_/d_s_* which depends entirely on the thermal properties of the substrate. However, it is the respective ratio’s of *C*, *K*, and *h* that determine the equilibration of temperature in the system. Since the temperature dependence of individual parameters is not generic (or known) for all materials, we restrict our analysis to few characteristic parametric choices, known in literature and covering the regimes of both slow and fast heat diffusion in the system. Mainly for the computational convenience, we use *C* = 0.03 (which corrensponds to the real-unit heat capacity of the film *c*_*f*_ = 0.15 mJ/cm^3^K, see Methods), *K* = 0.06 (with corresponding heat conductivity of the film in real units *k*_*f*_ = 1.33 mW/cmK, see Methods), and *h* = 2 × 10^−4^ (the real-unit heat transfer coefficient of the film *h*_*f*_ = 16.94 W/cm^2^K, see Methods)^34^,^35^,^36^,^37^. We note, however, that if only temperature-independent thermal parameters of the superconducting film close to *T*_*c*_ are taken into account, one can use Wiedemann-Franz law to estimate the dimensionless heat capacity *C* = 0.65 (*c*_*f*_ = 3.25 mJ/cm^3^K) and heat conductivity *K* = 0.06 (*k*_*f*_ = 1.33 mW/cmK) independently of the considered material. In reality the substrate influence can hardly be neglected, and for example, in NbN films considered in ref. 38 the effective heat capacity was *d*_*s*_*c*_*s*_/*d* + *c*_*f*_ = 2.4 mJ/cm^3^K, total heat conductivity *d*_*s*_*k*_*s*_/*d* + *k*_*f*_ = 1.1 mW/cmK, and *h*_*f*_ = 56.5 W/cm^2^K. Thus, for either set of parameters the thermalization time *τ*_*th*_ defined by the ratio *C*/*K* does not exceed 100*τ*_*GL*_(0), which is considerably shorter than the period *τ* of the thermal potential *V*_*T*_ used in this work. A case of *C* and *K* leading to a thermalization time *τ*_*th*_ exceeding the period of the pinning potential *τ* would lead to heat accumulation in the channel due to the ineffective heat removal via diffusion, causing full depletion of superconductivity (i.e. forming a permanent Josephson junction, and completely suppressing stroboscopic effects, which is not of interest in the present analysis). Additionally, we estimate the time needed for a vortex to cross the considered sample, *τ*_*cross*_, to be of the order of 1000*τ*_*GL*_(0). When *τ* is in the range (*τ*_*th*_, *τ*_*cross*_) effective heat removal is established, causing the distinct ON and OFF states in the channel, but there exists no synchronised vortex motion in the sample, and thus no characteristic dynamics is found. Only when 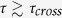 vortex motion in the channel will be completely governed by switching between ON and OFF states, causing the particular stroboscopic behavior of the condensate.

### Stroboscopic resonances and the link to vortex velocity

Here we point out general features of the stroboscopic regime governed by the switching between hot and cold states, in the presented setup. In the regime *τ* > *τ*_*cross*_ a synchronization between the vortex motion and the flashing channel will arise whenever the flashing period is long enough for an integer number of vortices to cross the sample along the depletion region, i.e. *τ* = *nτ*_*cross*_. During the synchronization, stroboscopic effect appears and manifests as recurring voltage drops in the *V*(*τ*). Quasi-periodic behavior of the *V*(*τ*) characteristics shown in Fig. 2 corroborates that, where in each consecutive stroboscopic state an additional vortex participates in the dynamics, relating the order of the resonance *n* exactly to the number of crossing vortices while the channel is open. In other words, the shift in the period *τ* between the subsequent resonances exactly corresponds to the average crossing time of one vortex, *τ*_*cross*_. This enables one to extract the average vortex velocity from the (experimentally accessible) voltage characteristics shown in Fig. 2 (as *W*/*τ*_*cross*_). During each resonance, the average voltage of the system follows a 1/*τ* functional dependence (specifically *V*_*n*_ = 2*πn*/*τ*), which is indicated by dotted lines in Fig. 2. To emphasize again, the drops in *V*(*τ*) exhibit periodicity which is exactly equal to the *τ*_*cross*_. Note that if the Eq. (1) is not taken into account (instantaneous heat diffusion and perfect heat removal in the system, as discussed in ref. 25 voltage resonances given in Fig. 2 are more narrow, with significantly shorter periodicity (~0.6τ cross). Fractional resonances observed in Fig. 2 (e.g. around *τ* = 600*τ*_*GL*_(0) and 1600*τ*_*GL*_(0), for *J* = 4.9 × 10^−3^*j*_0_, where *j*_0_(0) is current density unit, see Methods) originate from odd number of vortices traversing the channel over the pulse duration of 2*τ*. With prolonged flashing the occurrence of fractional resonances diminishes. By taking the above mentioned parameters of NbN, one can estimate the frequency (1/*τ*) presented in Fig. 2 to be in the range 4 GHz (shown stroboscopic resonances are expected to persist at lower frequencies as well) to 120 GHz^26^, which is still below the gap frequency of NbN (*ν*_*gap*_ ≈ 1 THz). The same values yield *τ*_*cross*_ ≈ 40 ps, vortex velocity 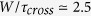 km/s, and observed voltage drops during the resonances ≈0.5 mV. The required pinning frequency to properly observe the resonances must be higher than 1/*τ*_*cross*_ (≈25 GHz). It is possible to lower 1/*τ*_*cross*_ by selecting a material with larger *τ*_*GL*_(0) or longer inelastic scattering time^25^. Alternatively, one can force vortices to travel longer distances by making the samples wider, thereby proportionally increasing *τ*_*cross*_. Additionally, we note here that all superconducting and vortex-related time-scales become longer if the temperature is lowered, without any qualitative changes to the reported stroboscopic behavior of the superconducting condensate, which is seemingly more convenient for practical realization of our predictions. However, since the specific heat, the thermal conductivity, and the heat transfer coefficient decrease with decreasing temperature, the heating becomes more localized - which may lead to heat accumulation so that periodic oscillations of the order parameter may no longer be achieved^39^. Note that this problem of local periodic heating and continuous cooling has been addressed in ref. 40 in the context of current driven phase slips in superconducting nanowires.

### Characteristic dynamics of the condensate

**Figure 3:**
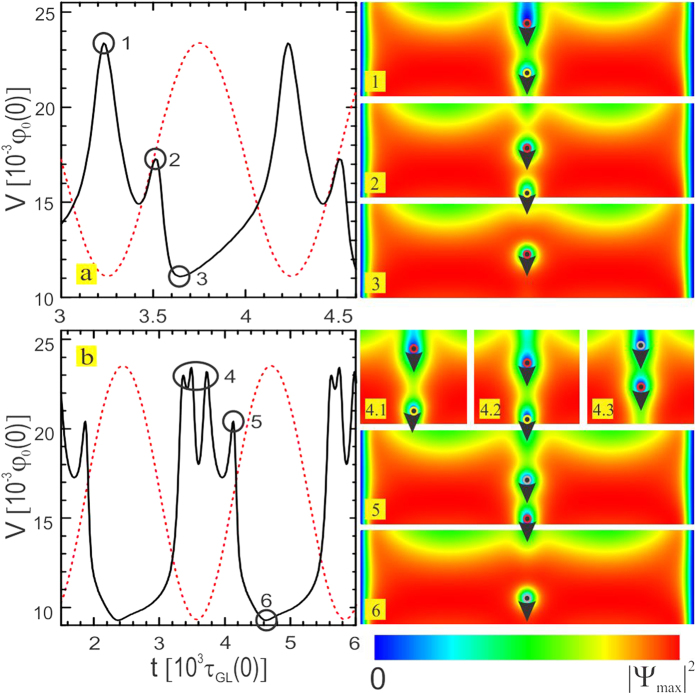
Temporal voltage response at the first and the second resonance. Dotted red lines show the profile of the flashing potential *V*_*T*_ in arbitrary units. (**a**) Instances (1)–(3) show characteristic condensate dynamics during one cycle of the first resonance, through corresponding Cooper-pair density snapshots: (1) new vortex enters the condensate as the channel is switched ON, (2) pre-existing vortex leaves the sample, (3) remaining vortex assumes position of the pre-existing vortex as the channel turns OFF. (**b**) During the second resonance, instances (4)-(6) show insight in one cycle of the condensate dynamics: (4) as the channel turns ON, a new vortex enters (4.1), pushing the pre-existing vortex to leave the sample (4.2), after which a second vortex entry occurs (4.3). While the channel is still ON, the first of the two remaining vortices leaves the condensate (5), and the channel turns OFF to trap one remaining vortex in the sample (6). The resulting periodicity of the voltage matches exactly the period of the channel oscillations.

Figure 3(a,b) present the real-time voltage *V*(*t*) for one value of the current density (*J* = 5.2 × 10^−3^*j*_0_), during the first [*τ* = 1100*τ*_*GL*_(0)] and the second [*τ* = 2260*τ*_*GL*_(0)] resonance, respectively (see Fig. 2). Instances (1)-(6) point out characteristic features of the superconducting dynamics through Cooper-pair density snapshots. The temporal dependence of the voltage in Fig. 3(a) (overlapped with the pinning intensity profile - dotted red line) correlates the vortex entry with a voltage peak at instance (1), just as the channel switches ON, pushing away the pre-existing vortex towards the sample edge. The subsequent exit of the pre-existing vortex produces a small peak in the voltage at instance (2). As the channel cools down one vortex remains in the sample, minimizes the voltage (3), and the cycle ends. One should easily notice that effectively, one vortex has crossed the sample during the flash of the potential, for an oscillation period corresponding to the first resonance. Dynamics during the second resonance in Fig. 3(b) shows a more complex voltage profile. Starting from channel ON, point (4) denotes time frame during which entrance of two new vortices in the sample occurs, as well as the exit of the pre-existing vortex [see snapshots (4.1), (4.2) and (4.3)]. At instance (5) the first of the two vortices that entered the sample leaves, with accompanying peak in the voltage. Finally, as the channel turns OFF, one remaining vortex is trapped, and the cycle ends. To facilitate the visualization and comparison of the first four resonances between the vortex dynamics and the pinning potential, we provide the animated data (entitled Supplementary-Animation1.gif) in the Supplementary Material.

### Vortex velocimetry

**Figure 4:**
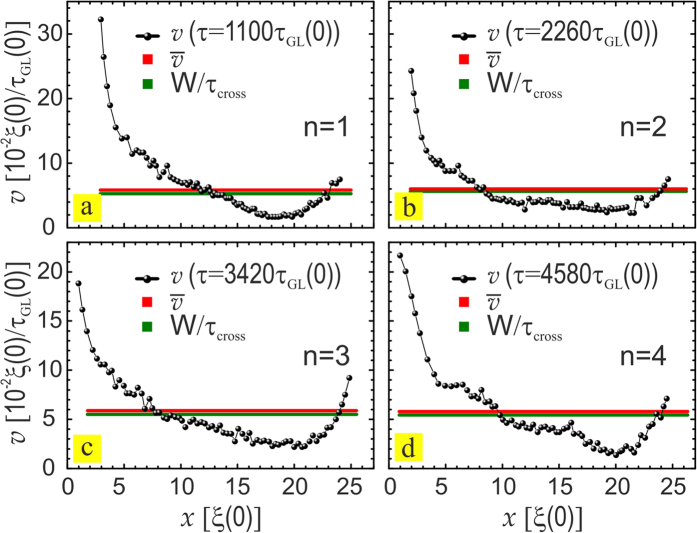
Vortex velocities. (**a–d**) Spatial distribution of the vortex velocity along the pinning channel during first four resonances. The black points show the instantaneous vortex velocities, directly measured in the simulations. Average vortex velocity (red line) compares well to the *W*/*τ*_*cross*_ (green line).

Typically in experiments the vortex velocity can only be reliably estimated at high magnetic fields, since vortex-vortex interaction dominates over vortex-pinning and therefore the velocity distribution function is very narrow^41^. As discussed above, stroboscopic resonances occur with periodicity that matches the crossing time of one additional vortex (*τ*_*cross*_) from which one can determine the average vortex velocity during the resonance. To verify this, in our numerical simulations we directly tracked the velocities of individual vortices as they pass along the channel, by developing a software capable of mapping the vortex trajectory in real time (shown in Fig. 4, for one value of applied current). The obtained velocity profiles resemble the velocity profiles measured in ref. 24. From this distribution of instantaneous vortex velocities we can compute the average vortex velocity (red line) which indeed matches the ratio of the channel length *W* and the corresponding *V*(*τ*) resonance period, *τ*_*cross*_ (green line), as predicted in the above analysis. Furthermore, by substituting the characteristic values of *ξ*(0) and *T*_*c*_ for different materials one can easily compare the vortex velocities to be expected. Using the parameters of Al (*ξ*(0) = 90 nm, *τ*_*GL*_(0) = 0.38 ps, *T*_*c*_ = 1.37 K^42^) and Pb (*ξ*(0) = 33 nm, *τ*_*GL*_(0) = 72 fs, *T*_*c*_ = 7.2 K^43^), one obtains the velocity multiplier ratios: 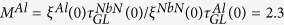 and 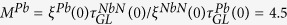, estimating expected velocity for Abrikosov vortices to be significantly larger than in NbN.

## Discussion

**Figure 5:**
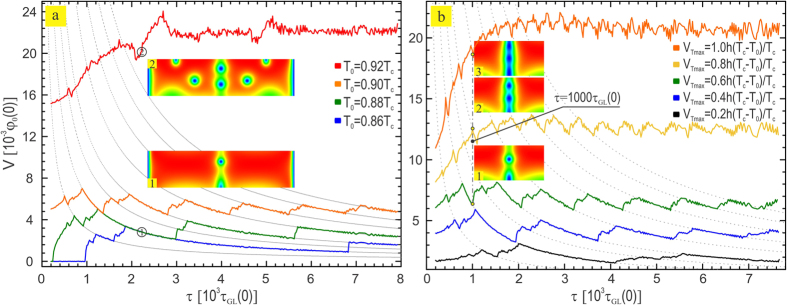
Influence of temperature on the resonant behavior. The resonant behavior in *V*(*τ*) for different bath temperatures (**a**), and for varied maximal temperature in the channel (**b**). (**a**) With increasing the bath temperature, a transition to a higher dissipative state may occur, where additional vortices move outside of the channel, thus creating additional harmonics that will smear out the average voltage from the 1/*τ* behavior [depicted by Cooper-pair density snapshots (1) for observable resonances and (2) for smeared resonances]. (**b**) By varying the maximal temperature in the channel, one may selectively switch between different vortex phases, from Abrikosov vortex, transitional state between Abrikosov and Josephson vortex, and to Josephson vortex [depicted by Cooper-pair density snapshots (1)–(3), respectively, at *τ* = 1000*τ*_*GL*_(0)]. The animations of different vortex species during one period of the characteristic dynamics of the condensate are provided in the Supplementary Materials (entitled Supplementary-Animation2.gif).

In what follows, we discuss the thermal effects related to the resonances and the vortex behavior through *V*(*τ*) characteristics shown in Fig. 5 for the same current used in Fig. 4, in the case when: (a) bath temperature is varied and (b) maximal temperature in the channel is varied. From Fig. 5(a) one can conclude that the resonant states are well preserved at low bath temperatures (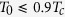), and that their behavior follows the trend exhibited in Fig. 2, where all vortices traverse the sample in a single line along the channel [snapshot (1) in Fig. 5(a)]. Especially for the lowest considered bath temperature *T*_0_ = 0.86*T*_*c*_, stroboscopic states appear at integer resonances, without exhibiting fractional resonances. Clear stroboscopic states persist up to a bath temperature exceeding 0.9*T*_*c*_, after which four additional crossing channels appear next to the pinning channel, along which vortices traverse with mismatched velocities. This excess vortex motion outside the pinning channel causes a jump to the higher dissipative state [snapshot (2) in Fig. 5(a), corresponding to *T*_0_ = 0.92*T*_*c*_]. In principle, stroboscopic resonances in the *V*(*τ*) characteristic still exist in the higher dissipative state, but are concealed and heavily smeared by the voltage harmonics produced from freely moving vortices out of the depletion region.

As a consequence of an external potential modulation, heat released in the channel varies, which affects the vortex motion in the channel. In Fig. 5(b) we show how the *V*(*τ*) characteristics change with increase of the thermal potential amplitude, 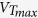. For a weak amplitude [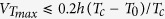] non-invasive regime is instated, where the vortex motion is barely affected, due to which the resonances are accompanied by very weak voltage oscillations. These oscillations become more visible as the amplitude is increased [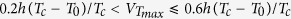], and we have resonant states comparable to results in Fig. 2. For further increased amplitude, more and more vortices pass through the channel during the resonance, so the *V*(*τ*) shifts to the left. For the selected value of the flashing period *τ* = 1000*τ*_*GL*_, we show that by simple tuning of 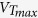 in the range [0.6*h*(*T*_*c*_ − *T*_0_)/*T*_*c*_, *h*(*T*_*c*_ − *T*_0_)/*T*_*c*_] one can switch between different resonant states and thereby manipulate the vortex velocity in the channel, thus entering the invasive mode. Moreover, the obtained results show that by varying the heat released in the channel, one can even controllably switch between different vortex phases and study them in more detail. Snapshots of the Cooper-pair density in Fig. 5(b) indicate that the vortex core deforms progressively with increasing heating, so that the found vortex phase at the same resonance for sequentially increasing 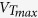 can change from the Abrikosov vortex (1), to Abrikosov-Josephson transition (2), and then Josephson vortex (3)^6^,^15^,^44^,^45^,^46^ [we provide animated data for different vortex phases in the Supplementary Material (entitled Supplementary-Animation2.gif)]. The accompanying change in *τ*_*cross*_ indicates that vortex velocity multiply increases during the transition between these vortex phases. Finally we emphasize that for any given applied magnetic field (of magnitude lower than *H*_*c*2_), one can tune either the current or the local temperature in order to switch between different resonances and study different types of vortices with core deformation.

To summarise, in this manuscript we presented the unique properties of the resistive state in a type II superconductor with a thermally induced flashing channel of depleted superconductivity. Stroboscopic synchronization between the vortex dynamics and the thermal oscillations in the channel is found and we directly link the shift between the subsequent resonances in the measured voltage versus the flashing period to the time needed for one additional vortex to traverse the channel. As a consequence, the voltage characteristics versus the period of the pinning potential provides a direct and reliable measure of the vortex velocity, unattainable otherwise, and that in a broad range of velocities, from slow Abrikosov vortices, to tenfold faster Josephson ones. Our velocimetry method therefore enables experimental confirmation of the limits of vortex velocity and the realistic characterization of the speed of the emerging vortex-based devices and technology^47^,^48^,^49^,^50^.

## Methods

In this work, the generalized time-dependent Ginzburg-Landau (TDGL)^2^,^31^ equation is used to describe the behavior of the superconducting condensate (with order parameter Ψ(**r**, *t*)) in the presence of applied magnetic field (***H***) (with vector potential (***A***)) and transport current density *J*, when time-dependent thermal pinning potential is present in the specimen:


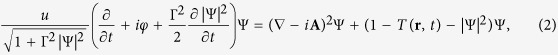


where *u* = 5.79 is the ratio of the relaxation times for amplitude and phase of the order parameter^2^,^31^,^51^, and the parameter Γ = 2*τ*_*in*_Δ(0)/*ħ* = 10 (*τ*_*in*_ is the inelastic phonon-electron scattering time) effectively characterizes the viscosity of the superconducting condensate to the vortex motion. This equation is coupled with the thermal balance equation [Eq. (1)] and with the equation for the electrostatic potential *φ*:


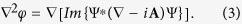


The generalized time-dependent Ginzburg-Landau theory used in this work was derived for the gapless superconductors containing high concentration of paramagnetic impurities. In the gapless case, the mechanism for pair-breaking is the strong inelastic electron-phonon scattering, and all the relevant quantities, such as Ψ and *A*, must relax on the time-scale longer than the inelastic phonon-electron scattering time (*τ*_*GL*_(0)/(1 − *T*/*T*_*c*_) ≫ *τ*_*in*_). Characteristic length over which these relaxation processes occur is 
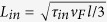
 (where *v*_*F*_ is the Fermi velocity, and *l* is the mean free path). This length is the distance over which an electric field can penetrate into the superconductor, and in the case of gapless superconductors it must be below the coherence length 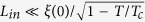. For majority of the materials, the previous conditions for slow temporal and spatial variation are satisfied at temperatures close to *T*_*c*_.

In our formalism, all distances are expressed in units of *ξ*(0), time in units of *τ*_*GL*_(0) = *πħ*/8*k*_*B*_*T*_*c*_*u*, and temperature in units of *T*_*c*_. Ψ is in units of Δ(0) = 4*k*_*B*_*T*_*c*_*u*^1/2^/*π*, *φ* in units of *φ*_0_(0) = *ħ*/e^*^τ_*GL*_(0), vector potential *A* is scaled to *A*_*s*_(0) = *H*_*c*2_(0)*ξ*(0) [*H*_*c*2_(0) = Φ_0_/2*πξ*^2^(0)], and current density to *j*_0_ = *σ*_*n*_*φ*_0_(0)/*ξ*(0), where *σ*_*n*_ is normal state conductivity. In Eq. (1) dimensionless heat capacity is obtained as 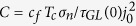, dimensionless heat conductivity as 
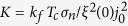
, while the dimensionless heat transfer coefficient is defined as 
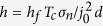
.

At the superconductor-vacuum (SV) boundary, the current density component perpendicular to the interface vanishes [**n**_*SV*_(∇ − *i***A**)Ψ = 0 and **n**_*SV*_∇*φ* = 0, where the ***n***_*SV*_ is the unit vector perpendicular to the surface of the SV boundary], and all heat is assumed to be transferred to the substrate (**n**_*SV*_∇*T* = 0). We consider metallic current leads (SN contacts), where the applied current transforms completely into normal current (−**n**_*SN*_∇*φ* = *J* and Ψ = 0, **n**_*SN*_ being the unit vector perpendicular to the surface of the SN boundary). There, the temperature is set to the critical temperature (*T*_*SN*_ = *T*_*c*_).

In all simulations, adaptive simulation time is taken, with an initial value of 5 × 10^4^*τ*_*GL*_(0), sufficiently long for a system to reach the dynamic equilibrium, and then extended by thirty temporal periods of the pinning potential (*t*_*sim*_ = 5 × 10^4^*τ*_*GL*_(0) + 30*τ*) for averaging of the outputs. The above equations are solved self-consistently in an iterative procedure, where the generalized time-dependent Ginzburg-Landau equation [Eq. (2)] is solved using a combination of Euler and Gauss-Seidel method^52^. We used the spectral Fourier method to solve the equation for the electrostatic potential, while the solver for the equation of thermal balance [Eq. (1)] was based the Crank-Nicolson scheme and Alternating Direction Implicit method.

## Additional Information

**How to cite this article**: Jelić, Ž.L. *et al*. Velocimetry of superconducting vortices based on stroboscopic resonances. *Sci. Rep*. **6**, 35687; doi: 10.1038/srep35687 (2016).

## Supplementary Material

Supplementary Information

Supplementary Animation 1

Supplementary Animation 2
