# Supplementary material for: Velocimetry of superconducting vortices based on stroboscopic resonances
Source: Sci Rep. 2016 Oct 24;6:35687. doi: 10.1038/srep35687 (PMC5075923; doi:10.1038/srep35687)
Supplement: Supplementary Information [file srep35687-s1.pdf]

Supplementary materials: Velocimetry of superconducting vortices based on stroboscopic resonances

Ž. L. Jelić,[1,2,*] M. V. Milošević,[2] and A. V. Silhanek[1]

[1]*Département de Physique, Université de Liège*

Aleé du 6-Août 19, B-4000 Liège, Belgium

[2]*Departement Fysica, Universiteit Antwerpen*

Groenenborgerlaan 171, B-2020 Antwerpen, Belgium

(Dated: June 28, 2016)

SUPPLEMENTARY ANIMATION 1: STROBOSCOPIC VOLTAGE RESONANCES.

As the Supplementary Material we present animations of the condensate behavior corresponding to the first four resonances during one period τ of the characteristic dynamics of the condensate (entitled Supplementary-Animation1.gif).

SUPPLEMENTARY ANIMATION 2: SWITCHING BETWEEN DIFFERENT VORTEX PHASES.

The animations of different vortex species during one period of the characteristic dynamics of the condensate are provided in this Supplementary Materials (entitled Supplementary-Animation2.gif).

* Correspondence to zeljko.jelic@ulg.ac.be
