# Supplementary figures and images for: Velocimetry of superconducting vortices based on stroboscopic resonances
Source: Sci Rep. 2016 Oct 24;6:35687. doi: 10.1038/srep35687 (PMC5075923; doi:10.1038/srep35687)

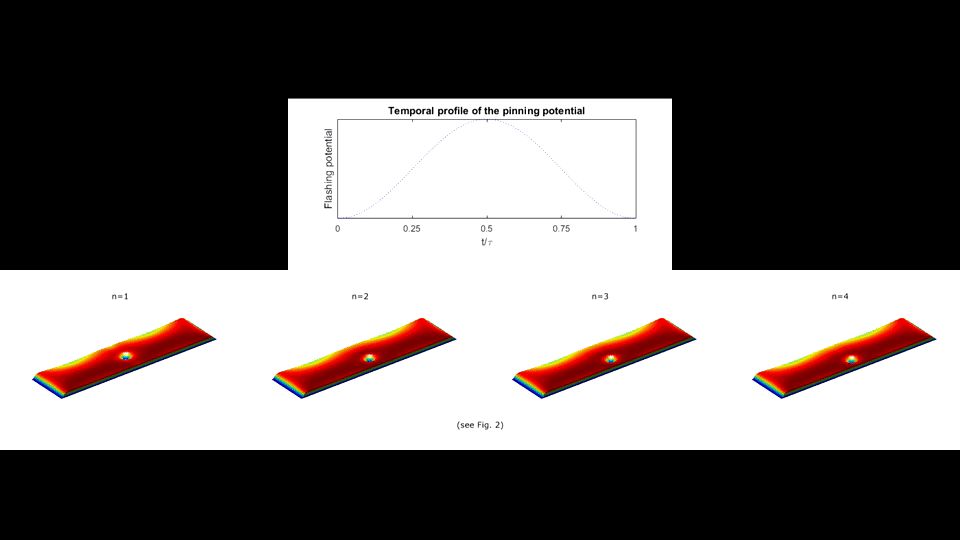

Supplement: Supplementary Animation 1 [file srep35687-s2.gif]

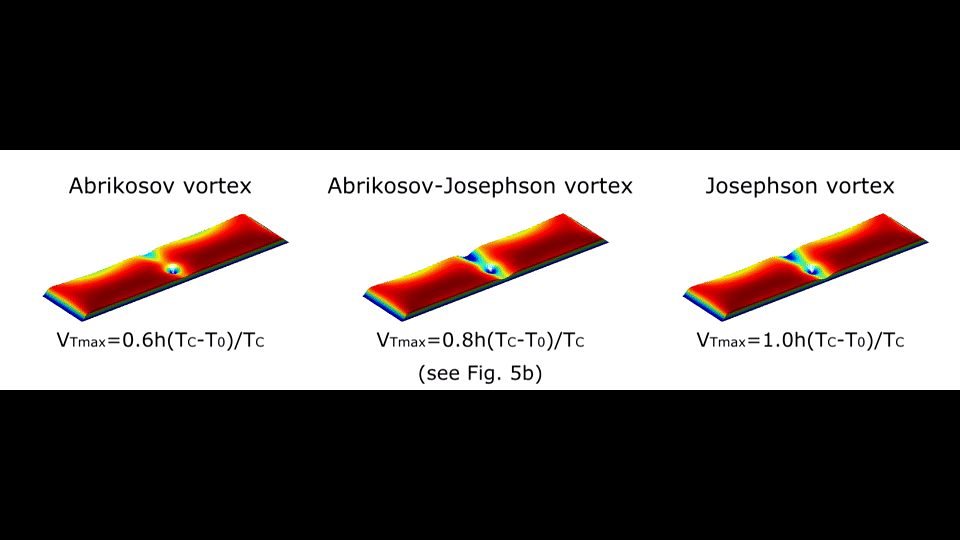

Supplement: Supplementary Animation 2 [file srep35687-s3.gif]
